# Unlocking the potential of approved drugs for the allosteric inhibition of tropomyosin-receptor kinase A using molecular docking and molecular dynamics studies

**DOI:** 10.3389/fchem.2023.1205724

**Published:** 2023-06-07

**Authors:** Rua M. Mukhtar, Nihal Abdelmoniem, Hisham A. Elrufaie, Alaa Edris, Hiba Ghaboosh, Mohanad A. Mahgoub, Elrashied A. E. Garelnabi, Wadah Osman, Asmaa E. Sherif, Ahmed Ashour, Kholoud F. Ghazawi, Waad A. Samman, Aisha A. Alhaddad, Rawan Bafail, Sabrin R. M. Ibrahim, Gamal A. Mohamed, Abdulrahim A. Alzain

**Affiliations:** ^1^ Department of Pharmaceutical Chemistry, Faculty of Pharmacy, University of Gezira, Gezira, Sudan; ^2^ Department of Pharmaceutics, Faculty of Pharmacy, University of Gezira, Gezira, Sudan; ^3^ Department of Pharmaceutical Chemistry, Faculty of Pharmacy, University of Khartoum, Khartoum, Sudan; ^4^ Department of Pharmacognosy, Faculty of Pharmacy, Prince Sattam Bin Abdulaziz University, Al-kharj, Saudi Arabia; ^5^ Department of Pharmacognosy, Faculty of Pharmacy, University of Khartoum, Khartoum, Sudan; ^6^ Department of Pharmacognosy, Faculty of Pharmacy, Mansoura University, Mansoura, Egypt; ^7^ Clinical Pharmacy Department, College of Pharmacy, Umm Al-Qura University, Makkah, Saudi Arabia; ^8^ Department of Pharmacology and Toxicology, College of Pharmacy, Taibah University, Al-Madinah Al-Munawwarah, Saudi Arabia; ^9^ Department of Pharmaceutics and Pharmaceutical Technology, College of Pharmacy, Taibah University, Medina, Saudi Arabia; ^10^ Preparatory Year Program, Department of Chemistry, Batterjee Medical College, Jeddah, Saudi Arabia; ^11^ Department of Pharmacognosy, Faculty of Pharmacy, Assiut University, Assiut, Egypt; ^12^ Department of Natural Products and Alternative Medicine, Faculty of Pharmacy, King Abdulaziz University, Jeddah, Saudi Arabia

**Keywords:** cancer, tropomyosin-receptor kinase A, repurposing, molecular docking, molecular dynamics, drug discovery, health and wellbeing

## Abstract

Tropomyosin-receptor kinase A (TrkA) is the primary isoform among the tropomyosin-receptor kinases that have been associated with human cancer development, contributing to approximately 7.4% of all cancer cases. TrkA represents an attractive target for cancer treatment; however, currently available TrkA inhibitors face limitations in terms of resistance development and potential toxicity. Hence, the objective of this study was to identify new allosteric-approved inhibitors of TrkA that can overcome these challenges and be employed in cancer therapy. To achieve this goal, a screening of 9,923 drugs from the ChEMBL database was conducted to assess their repurposing potential using molecular docking. The top 49 drug candidates, exhibiting the highest docking scores (−11.569 to −7.962 kcal/mol), underwent MM-GBSA calculations to evaluate their binding energies. Delanzomib and tibalosin, the top two drugs with docking scores of −10.643 and −10.184 kcal/mol, respectively, along with MM-GBSA dG bind values of −67.96 and −50.54 kcal/mol, were subjected to 200 ns molecular dynamic simulations, confirming their stable interactions with TrkA. Based on these findings, we recommend further experimental evaluation of delanzomib and tibalosin to determine their potential as allosteric inhibitors of TrkA. These drugs have the potential to provide more effective and less toxic therapeutic alternatives. The approach employed in this study, which involves repurposing drugs through molecular docking and molecular dynamics, serves as a valuable tool for identifying novel drug candidates with distinct therapeutic uses. This methodology can contribute to reducing the attrition rate and expediting the process of drug discovery.

## 1 Introduction

Tropomyosin-receptor kinases (Trks), a subfamily of the protein kinase superfamily, belong to the receptor tyrosine kinases and consist of three isoforms: TrkA, TrkB, and TrkC. These isoforms function as receptors for the neurotrophin family, which includes high-affinity growth factors such as nerve growth factor (NGF), which binds to TrkA, brain-derived neurotrophic factor (BDNF) and neurotrophin-4/5 (NT4/5), which bind to TrkB, and neurotrophin-3 (NT3), which binds to TrkC (Wang et al., 2009).

Previous experimental research has provided cumulative data indicating the involvement of Trks in the pathogenesis of a diverse range of human cancers, which has led to their recognition as promising targets for cancer treatment (Wang et al., 2009; Alam et al., 2017). TrkA, in particular, is considered oncogenic, with mounting evidence pointing to its overexpression and involvement in cancer development (Griffin et al., 2020). It is the most common isoform of Trks and is frequently associated with gene mutations or fusions, which result in the formation of oncogenes responsible for approximately 7.4% of all human cancer cases (Guo et al., 2022).

Trk inhibitors can be classified into four categories based on their binding interactions: type I, type II, type III, and type IV (Wu et al., 2015). Type I inhibitors are ATP-competitive and bind to the ATP active site. Type II inhibitors, on the other hand, are ATP non-competitive and exhibit pseudo-competitive binding kinetics by extending into a deep hydrophobic pocket within the ATP-binding site. Type III inhibitors are allosteric and bind adjacent to the ATP-binding site, while type IV inhibitors bind to regions other than the kinase domain of the protein (Yan et al., 2019). Type II inhibitors offer higher selectivity than type I inhibitors, but their large molecular size limits their druggability. However, both type I and type II inhibitors face challenges due to the emergence of secondary mutations in the ATP active site of Trks, particularly TrkA. Type III and type IV inhibitors provide isoform selectivity, although the effectiveness of type IV inhibitors as anticancer agents remains uncertain (Yan et al., 2019). Therefore, this study aims to identify allosteric TrkA selective inhibitors (type III) to overcome the existing limitations of TrkA inhibitors. These inhibitors could potentially be used in the management of various cancers associated with TrkA activation, such as lung, breast, cervix, thyroid, and oral cavity cancers (Lagadec et al., 2009; Sasahira et al., 2013; Faulkner et al., 2018; Gao et al., 2018; Faulkner et al., 2020).

Similar to other RTKs, TrkA comprises three domains: an extracellular domain responsible for ligand binding, a transmembrane domain, and an intracellular catalytic domain (Amatu et al., 2019). The region between the transmembrane domain and the catalytic domain, known as the juxtamembrane (JM) region, consists of approximately 60 residues. Interestingly, this region exhibits approximately 36% similarity with TrkB and 40% similarity with TrkC (Su et al., 2017; Furuya et al., 2017). In the inactive state, the Asp–Phe–Gly (DFG) motif of TrkA’s activation loop adopts an “out” conformation. This conformation is stabilized by edge-to-face interactions involving three phenylalanine residues: DFG motif Phe669, gatekeeper Phe589, and back pocket Phe575. Together, they form a unique FFF motif, along with the Leu564 residue in the *α*-C helix and the JM region. This combination generates an allosteric site adjacent to the ATP-binding site of TrkA (Bagal et al., 2019). X-ray crystallography studies have revealed that this allosteric site binds type III inhibitors, effectively maintaining TrkA in an inactive conformation (Simard et al., 2009; Heinrich et al., 2010). Therefore, this study leverages the knowledge of this allosteric site to identify type III TrkA inhibitors, utilizing its potential for modulating TrkA activity.

The process of drug discovery and development is known for its high attrition rate, involving significant time, cost, and effort, making the introduction of a new drug to the market a challenging endeavor (Mohammed et al., 2022; Gowtham et al., 2022). In the field of cancer research, the strategy of drug repositioning or repurposing has gained widespread application. This strategy involves repurposing approved or investigational drugs for new indications that were not initially intended for their use (Pushpakom et al., 2018; Gazerani, 2019; Omer et al., 2022). By leveraging existing drugs, the drug repositioning approach significantly reduces the time required for the drug discovery process by 3–5 years, lowers costs by $0.3 billion, and reduces failure rates in the later stages of development. This is because the drugs being investigated have already demonstrated sufficient safety profiles, enabling them to swiftly enter phases II and III of clinical trials (Fu et al., 2022; Issa et al., 2021).

Computational techniques play a vital role in drug repurposing, encompassing various approaches, such as molecular docking, genetic association, pathway mapping, data mining, and signature matching (Fu et al., 2022).

In this study, molecular docking coupled with MM-GBSA calculations and molecular dynamics (MD) simulations were employed to investigate drugs from the ChEMBL database. The aim of this study was to assess their potential for repurposing as drug candidates for cancer treatment, specifically targeting the TrkA allosteric site.

## 2 Materials and methods

All *in silico* studies, with the exception of the molecular dynamics (MD) simulations, were conducted using Maestro v12.8 from Schrödinger. The MD simulations were performed using Academic Desmond v6.5 by D.E. Shaw Research.

### 2.1 Protein and ligand preparation

The crystallographic structure of TrkA, along with the co-crystallized ligand (PDB ID: 6D20) (Bagal et al., 2019), was obtained from the Protein Data Bank (PDB) (https://www.rcsb.org/). To prepare the TrkA structure for subsequent calculations, a three-step processing procedure was performed using the Protein Preparation Wizard in Maestro.

In the first step, basic adjustments were made to the protein structure, including assigning bond orders, adding hydrogen atoms to those that were missing, creating zero-order bonds for metals and disulfide bonds, converting selenomethionines to methionines, filling in missing side chains and loops, removing water molecules beyond 5.00 Å from heterogroups, and generating potential ionization states of heteroatoms at a pH of 7 ± 2.

The second step involved optimizing hydrogen bonds and assigning orientations to the crystalized water molecules. The protonation states of the residues were also determined using the PROPKA tool at a pH of 7.0.

Finally, the third step involved restrained minimization, which was performed using the OPLS4 force field (AbdElmoniem et al., 2023). This step aimed to achieve a more stable and energetically favorable conformation of the TrkA structure for subsequent calculations.

We downloaded the drugs library from the ChEMBL database at https://www.ebi.ac.uk/chembl/. Specifically, we focused on the category of drug molecules. Within this category, we narrowed our focus to small molecules, which encompass various types, such as FDA-approved, world-approved, and investigational compounds. In total, we selected 9,923 small molecules from this category, representing a diverse range of therapeutic classes. To prepare the library for further analysis, the LigPrep tool in Maestro was employed (Alzain and Elbadwi, 2021). LigPrep not only generated low-energy three-dimensional structures for the input compounds but also produced multiple output structures for each compound. This was achieved by considering various factors, such as possible ionization states, tautomers, and stereoisomers. The LigPrep process was executed with the default settings, ensuring comprehensive exploration of the chemical space represented by the drug library.

### 2.2 Grid generation and molecular docking

The prepared protein structure underwent the receptor grid generation process, a crucial step for ligand docking. This process generated a grid file representing the site on the receptor where the ligand docking would occur. The receptor grid generation panel in Maestro was utilized to configure the grid generation job (Mohamed et al., 2022). The ligand molecule that bound to the TrkA allosteric site was identified and excluded from the grid generation process. This step helped define the position and size of the allosteric site surrounding the ligand. The van der Waals scaling and other options in the panel were kept at their default settings, and the grid generation process was initiated.

To evaluate the strength and affinity of the compounds toward the target’s allosteric site, the prepared library underwent molecular docking using the ligand docking panel in the Glide tool of Maestro (Elbadwi et al., 2021; Alzain et al., 2022). Initially, the library was subjected to a high-throughput virtual screening (HTVS) mode. The top compounds were then filtered based on their docking scores and subsequently subjected to an extra-precision (XP) docking mode. This multi-step docking process enabled the identification of potential compounds that exhibited favorable binding characteristics and affinity for the TrkA allosteric site. As a reference, the co-crystallized ligand was also docked onto the allosteric site.

### 2.3 MM-GBSA calculations

The ligand that poses with the best docking scores were selected and subjected to free-binding energy calculations using the molecular mechanics-generalized born surface area (MM-GBSA) method. These calculations were performed using the Prime tool in Maestro. The MM-GBSA method was utilized to estimate the free-binding energy of the ligand–receptor complex. The specific equation employed in these calculations to determine the free-binding energy is as follows:

$$∆E=E_{c}–E_{R}–E_{L},$$

where ∆E is the free-binding energy, E_c_ is the ligand–receptor complex energy, E_R_ is the receptor energy, and E_L_ is the ligand energy (Obubeid et al., 2022). The force field and the solvent model were set to be OPLS4 and VSGB, respectively.

### 2.4 MD simulation

The two ligand–protein complexes with the best docking scores and free-binding energy, as well as the co-crystallized ligand, were chosen for the molecular dynamics (MD) simulation study. The MD simulations were conducted using Desmond software (Alzain et al., 2022).

Prior to the simulation process, the biological system was set up using the System Builder panel in Desmond. This involved solvating the ligand–protein complexes with 12,437 TIP3P water molecules in an orthorhombic-shaped box with dimensions of 10 × 10 × 10 Å. Additionally, 51.167 mM of Na^+^ ions (with a total charge of +35) and 51.167 mM of Cl^−^ions (with a total charge of −35) were added as salt to maintain the system’s electrostatic neutrality. The OPLS4 force field was employed to minimize the energy of the system. Subsequently, the system underwent equilibration in two ensembles: isothermal–isochoric (NVT) and isothermal–isobaric (NPT). During the NVT ensemble, the system’s temperature was maintained at 300 K, while during the NPT ensemble, both temperature and atmospheric pressure (1 bar) were kept constant. The Nose–Hoover chain thermostat and the Martyna–Tobias–Klein barostat methods were employed to maintain the desired temperature and pressure conditions, respectively. The trajectory was recorded at a 100-ps interval, resulting in a total of 2,000 frames.

The analysis of the simulation results was performed using the Simulation Interaction Diagram tool provided by Desmond.

## 3 Results and discussion

**FIGURE 1:**
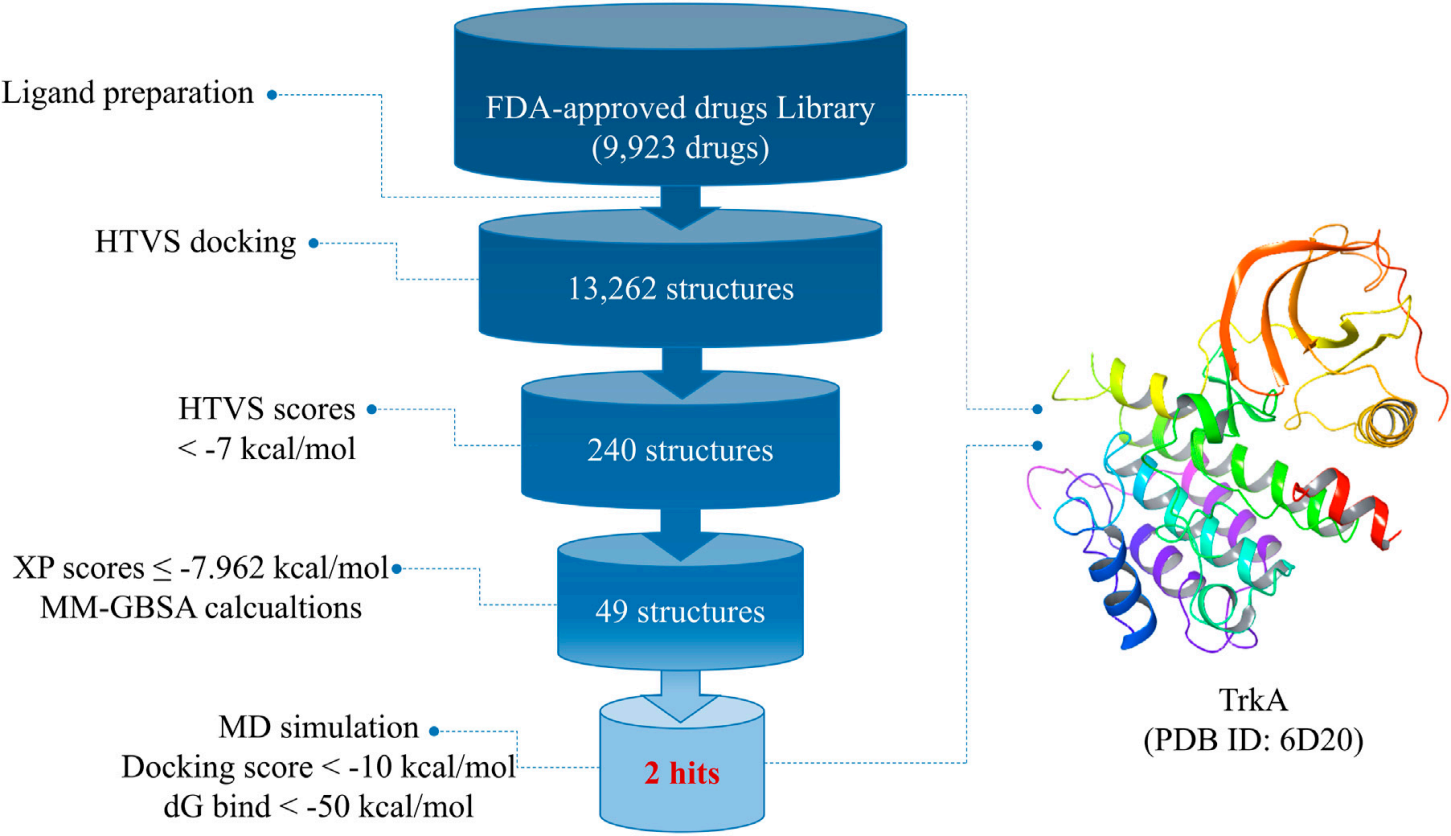
Overall work summary.

Figure 1 provides an overview of the research workflow, which employed various *in silico* methods to investigate the potential discovery of drug candidates from FDA-approved drugs for the inhibition of TrkA protein kinases. In the drug discovery process, molecular docking and molecular dynamics simulations play crucial roles in understanding ligand–receptor interactions. These computational approaches are particularly valuable for developing medications targeting new and challenging diseases such as cancer. The research also utilized virtual screening and drug repurposing strategies to identify potential drug candidates. By leveraging these *in silico* techniques, the study aimed to uncover promising candidates for TrkA inhibition.

### 3.1 Molecular docking

The molecular docking analysis was performed using the Glide module of Schrödinger. Glide is a powerful tool that accurately determines the positions and orientations of ligands within the active site of the receptor, providing valuable information on the compounds’ affinity and activity (Alzain and Elbadwi, 2021; Meng et al., 2011). It employs various scoring functions to rank and select the best poses for further analysis (Friesner et al., 2004). Glide offers three levels of docking methodologies: high-throughput virtual screening (HTVS), standard precision (SP), and extra-precision (XP). Each methodology differs in accuracy, with HTVS being the fastest but least accurate and XP being the most accurate but time-consuming. The docking time for screening one compound ranges from 2 s (HTVS) to 2 min (XP) (Eltaib and Alzain, 2022). These methodologies can be used sequentially to efficiently filter a large number of compounds. Furthermore, the molecular docking performed by Glide sets the stage for predicting the free-binding energy using methods such as MM-GBSA calculations.

After preparing the library of FDA-approved drugs (9,923 molecules) using the LigPrep tool, we generated a total of 23,334 conformers and tautomers. These compounds were subjected to molecular docking against the TrkA allosteric site using the high-throughput virtual screening (HTVS) mode. Among them, 230 structures with docking energies below −7 kcal/mol were identified as potential ligands. Since this number was manageable for further analysis, these 230 structures were directly subjected to molecular docking using the extra-precision (XP) mode, bypassing the standard precision (SP) level. Among the XP docking results, 49 structures were selected based on their docking scores, which ranged from −11.569 to −7.962 kcal/mol, for subsequent free-binding energy prediction.

### 3.2 MM-GBSA calculations

Docking results provide insights into whether ligands bind to the active site of the target protein. However, to determine if this binding is stable and capable of eliciting a response, it is crucial to assess the free-binding energy of the receptor–ligand complex (Lyne et al., 2006). Therefore, the top 49 structures from the docking results were further analyzed using the MM-GBSA method, which accounts for the solvent’s influence on the ligand–protein complex binding. For comparison, the co-crystallized ligand of TrkA was also subjected to XP and MM-GBSA calculations as a reference.

Among the 49 structures, nine drugs were selected based on their docking scores (<−9) and MM-GBSA dG bind energies (<−50 kcal/mol) for further investigation (Table 1). As shown in Table 1, none of the nine chosen drugs achieved better docking scores or MM-GBSA dG bind energies than the reference compound, which had a docking score of −10.689 and MM-GBSA dG bind of −105.51 kcal/mol. However, the results are considered satisfactory since the difference in docking scores between the selected compounds and the reference is minimal, and their MM-GBSA dG bind energies are highly favorable.

**TABLE 1:** Docking scores and MM-GBSA dG bind energies of the nine selected best ligand poses and the reference bound to TrkA allosteric site.

Compound name	Docking score kcal/mol	MM-GBSA dG bind kcal/mol
Delanzomib	−10.643	−67.96
Tibalosin	−10.184	−50.54
Vismodegib	−9.948	−53.56
Hexoprenaline	−9.666	−62.39
Merestinib	−9.342	−64.24
Etanterol	−9.146	−55.76
Ractopamine	−9.117	−53.23
Primidolol	−9.084	−54.35
Cliropamine	−9.022	−52.04
TrkA–ligand	−10.689	−105.51

Among the chosen drugs, delanzomib and tibalosin, with docking scores of −10.643 and −10.184 kcal/mol and MM-GBSA dG bind energies of −67.96 and −50.54 kcal/mol, respectively, stood out as representatives for further analysis of their interaction patterns.

### 3.3 Ligand–residue interaction analysis

**FIGURE 2:**
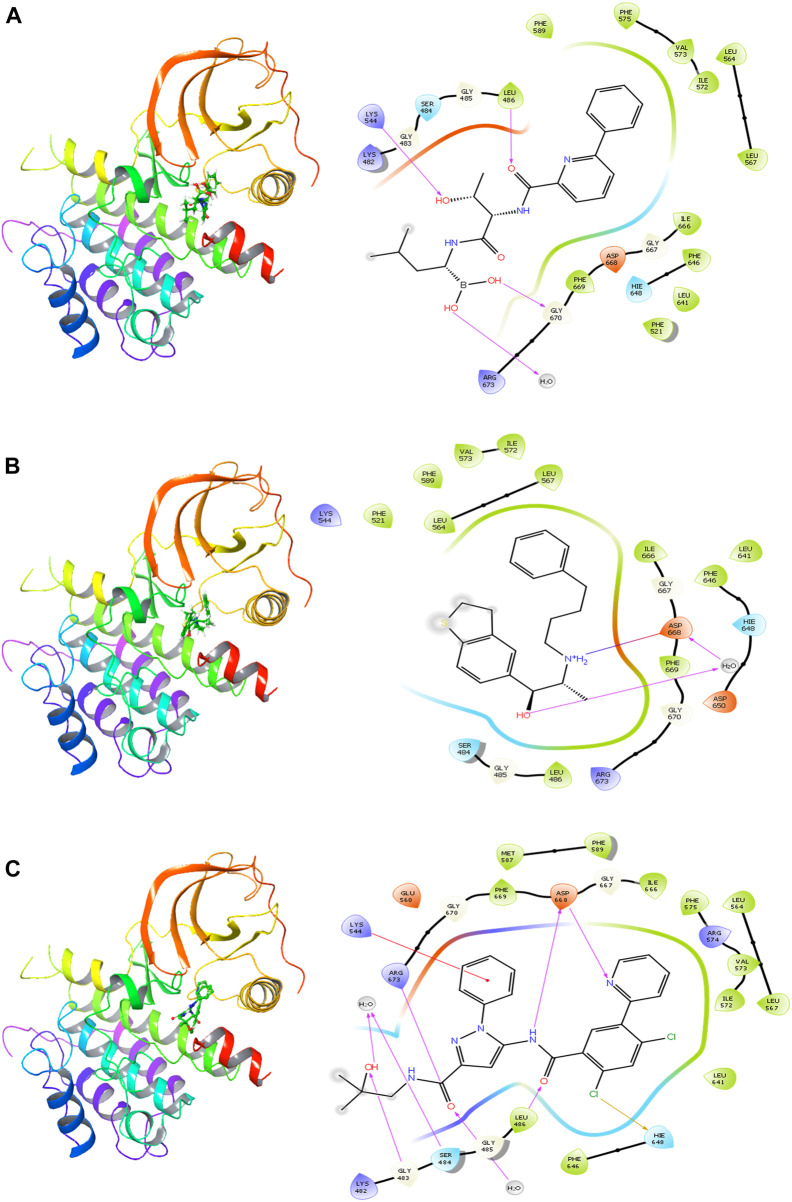
2D and 3D interactions of the best three hits with the TrkA allosteric site (PDB ID: 6D20) using Glide software. **(A)** Delanzomib. **(B)** Tibalosin. **(C)** Reference.

The delanzomib/TrkA complex exhibited three hydrogen bonds with LEU486, LYS544, and GLY670 residues, along with hydrophobic contacts with LEU486, PHE521, LEU564, LEU567, ILE572, VAL573, PHE575, PHE589, LEU641, PHE646, ILE666, and PHE669 (Table 2; Figure 2A), while the tibalosin/TrkA complex formed one hydrogen bond with ASP668, one salt bridge with ASP668, and hydrophobic contacts with LEU486, PHE521, LEU564, LEU567, PHE589, ILE572, VAL573, LEU641, PHE646, ILE666, and PHE669 (Table 2; Figure 2B). On the other hand, the reference/TrkA complex exhibited six hydrogen bonds, involving GLY483, SER484, LEU486, ARG673, and ASP668 residues. Additionally, it formed one halogen bond with HIE648, one pi–cation interaction with LYS544, and hydrophobic contacts with LEU486, LEU564, LEU567, MET587, PHE589, ILE572, VAL573, PHE575, LEU641, PHE646, ILE666, and PHE669 (Table 2; Figure 2C).

**TABLE 2:** Ligand–residue interactions of delanzomib and tibalosin at the TrkA allosteric site.

Compound name	H-bond	Salt bridge	Hydrophobic interaction	Other interaction
Delanzomib	LEU486, LYS544, and GLY670	-	LEU486, PHE521, LEU564, LEU567, ILE572, VAL573, PHE575, PHE589, LEU641, PHE646, ILE666, and PHE669	Polar interaction: SER484 and HIE648

				Charged negative: ASP668

				Charged positive: LYS482, LYS544, and ARG673

Tibalosin	ASP668	ASP668	LEU486, PHE521, LEU564, LEU567, PHE589, ILE572, VAL573, LEU641, PHE646, ILE666, and PHE669	Polar interaction: SER484 and HIE648

				Charged negative: ASP668 and ASP650

				Charged positive: LYS544 and ARG673

Reference	GLY483, SER484, LEU486, ASP668, and ARG673	-	LEU486, LEU564, LEU567, MET587, PHE589, ILE572, VAL573, PHE575, LEU641, PHE646, ILE666, and PHE669	Polar interaction: SER484 and HIE648

				Charged negative: GLU560 and ASP668

				Charged positive: LYS482, LYS544, ARG574, and ARG673

				Halogen bond: HIE648

				Pi–cation: LYS544


The ligand–residue interaction analysis provides insights into the differences observed in the docking scores and MM-GBSA dG bind energies among the reference, delanzomib, and tibalosin complexes. The reference compound showed the highest number of hydrogen bonds (6), followed by delanzomib (3) and Tibalosin (1). This highlights the importance of hydrogen bond interactions in contributing to the binding affinity of ligands (Klebe and Böhm, 1997; Chen et al., 2016; Anandan et al., 2022).

It is worth noting that the interactions observed between delanzomib, tibalosin, and specific residues in the TrkA protein align with findings from previous research articles investigating small molecules as TrkA allosteric inhibitors (Furuya et al., 2017; Su et al., 2017; Bagal et al., 2019; Subramanian et al., 2019; Guo et al., 2022). For instance, ASP668 has been reported to form hydrogen bonds with several top inhibitors discovered by different scientific groups. In the case of tibalosin, ASP668 interacts with the hydroxyl group and the amino group of the (2R)-2-[(4-phenylbutyl)amino]propan-1-ol moiety through hydrogen bond and salt bridge interactions, respectively. Although delanzomib does not form a hydrogen bond with ASP668, it establishes hydrogen bond interactions with LEU486, LYS544, and GLY670, which have also been documented in previous studies (Furuya et al., 2017; Bagal et al., 2019; Subramanian et al., 2019; Guo et al., 2022).

Furthermore, previous studies by Guo et al. (2022) and Bagal et al. (2019)*.* have emphasized the significance of hydrophobic interactions with LYS544, LEU564, and PHE589 in TrkA allosteric inhibitors. In the case of delanzomib and tibalosin, both compounds form hydrophobic contacts with LEU564 and PHE589 and establish a charged positive contact with LYS544.

Additionally, a thorough review of the identity and previous records of delanzomib and tibalosin was conducted to determine if any documented activity or correlation with cancer treatment exists.

Delanzomib is an orally active boronate-based proteasome inhibitor that specifically targets the chymotrypsin-like activity of the proteasome (Dolloff, 2015). While information regarding delanzomib’s impact on bone remodeling is limited, one study has explored its effects on osteoclasts (Zangari and Suva, 2016). In a study by Mopei et al., delanzomib demonstrated promising efficacy and antimutagenic properties in human multiple myeloma cell lines and patient-derived cells (Wang et al., 2019). Additionally, delanzomib has shown significance in the treatment of renal cell carcinoma (RCC). When combined with ritonavir, these two drugs exhibited synergistic effects in suppressing colony formation and inhibiting the growth of renal cancer (Isono et al., 2018).

Tibalosin, a phenylethylamine derivative, has been shown to reduce arterial pressure in hypertension animal models (Staessen et al., 1983). It exhibits potent antihypertensive effects; however, side effects prevent its clinical use at a daily dose of 150 mg. Combination therapy with a beta-adrenoceptor-blocking medication appears to be more effective in treating hypertension than thiazide therapy alone (Staessen et al., 1986). Currently, there is no available data linking tibalosin to cancer or its anticancer properties.

In conclusion, based on the docking, MM-GBSA, and interaction pattern analysis results, delanzomib and tibalosin demonstrate promise as TrkA inhibitors. Delanzomib has reported an anticancer activity, while tibalosin presented a potential anticancer activity as a TrkA inhibitor, which is being reported for the first time in this study. Furthermore, both drugs were subjected to a 200 ns molecular dynamics (MD) simulation study to further explore their behavior and interactions.

### 3.4 MD simulations

The previous techniques employed rigid structures for proteins and ligands, whereas molecular dynamics (MD) simulation takes into account the conformational changes in the receptor and ligand. MD simulation provides a more realistic representation of the dynamic behavior occurring under physiological conditions, allowing for a thorough investigation of the complex’s stability, flexibility, and binding interactions (Kumar et al., 2019; Jordaan et al., 2020; Aghajani et al., 2022; Gowtham et al., 2023).

In this study, MD simulations were conducted for the complexes of the two best compounds, delanzomib and tibalosin, with TrkA, as well as the reference structure (the co-crystallized ligand of 6D20). The analyzed data include the root mean square deviation (RMSD), the root mean square fluctuation (RMSF), and the protein–ligand contacts observed during the 200 ns simulation.

**FIGURE 3:**
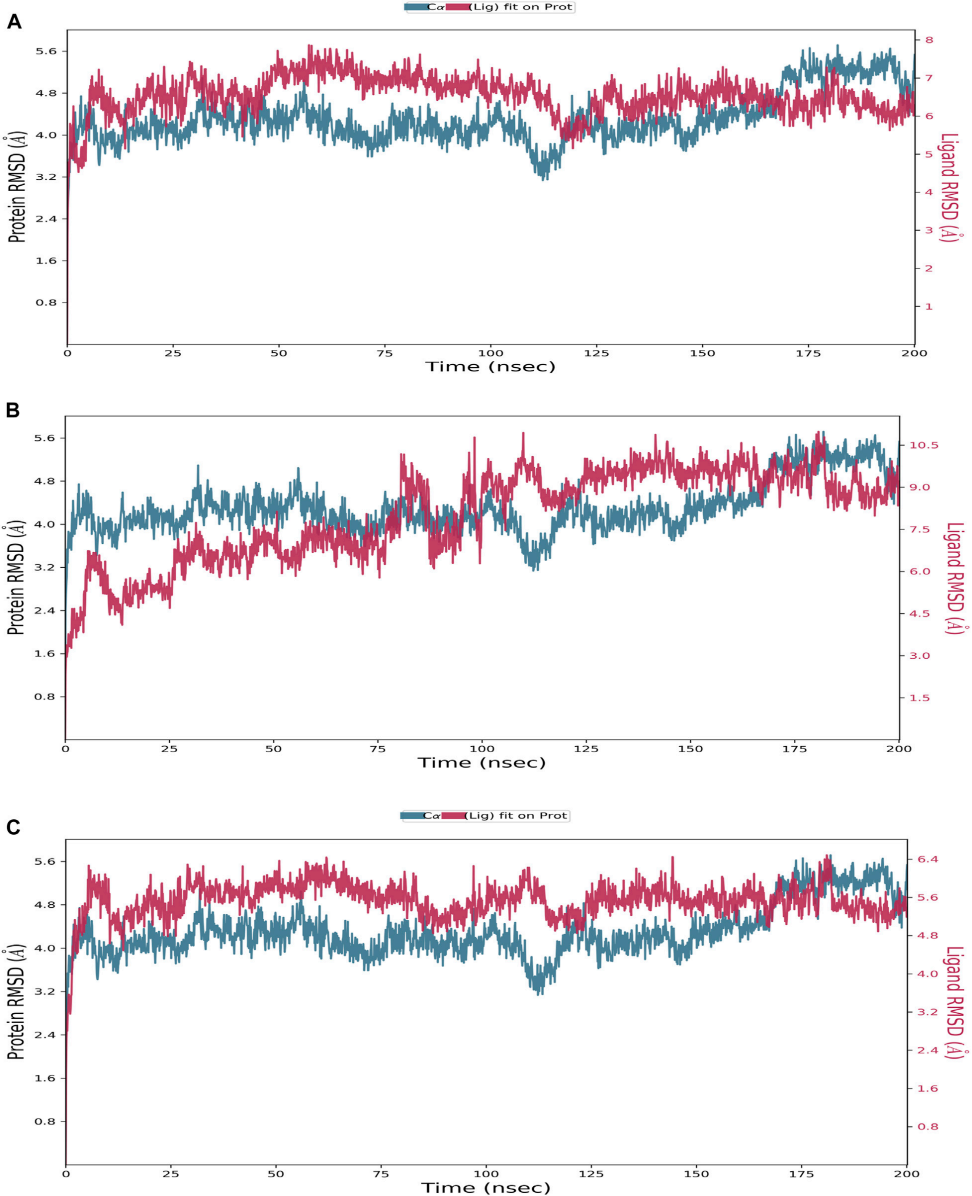
Protein–ligand RMSD plot of the top three compounds and the reference complexed with the TrkA allosteric site (PDB ID: 6D20) during 200 ns molecular dynamics simulation using Desmond software. **(A)** Delanzomib. **(B)** Tibalosin. **(C)** Reference.

Starting with the RMSD analysis of the protein’s Cα atoms (Figure 3), it can be observed that the protein exhibited a similar pattern of deviations with an average RMSD of 4.31 Å when complexed with the two compounds and the reference. This average RMSD value is relatively compatible, as an RMSD of 1–3 Å is generally acceptable for small globular proteins (Obubeid et al., 2022). It indicates the overall stability of the TrkA–ligand complexes. Delanzomib exhibited the lowest range of RMSD values, indicating greater stability than tibalosin and the reference. Delanzomib also showed minimal fluctuations along the simulation duration, with an average ligand RMSD of 2.81 Å. It is worth noting that the behavior of delanzomib closely resembled that of the reference.

On the other hand, tibalosin initially exhibited high fluctuations during the first 100 ns of the simulation, but its behavior became more similar to the reference in the second half of the simulation. Although tibalosin showed higher fluctuations, its average ligand RMSD of 1.63 Å was the smallest among delanzomib (2.81 Å) and the reference (2.61 Å).

**FIGURE 4:**
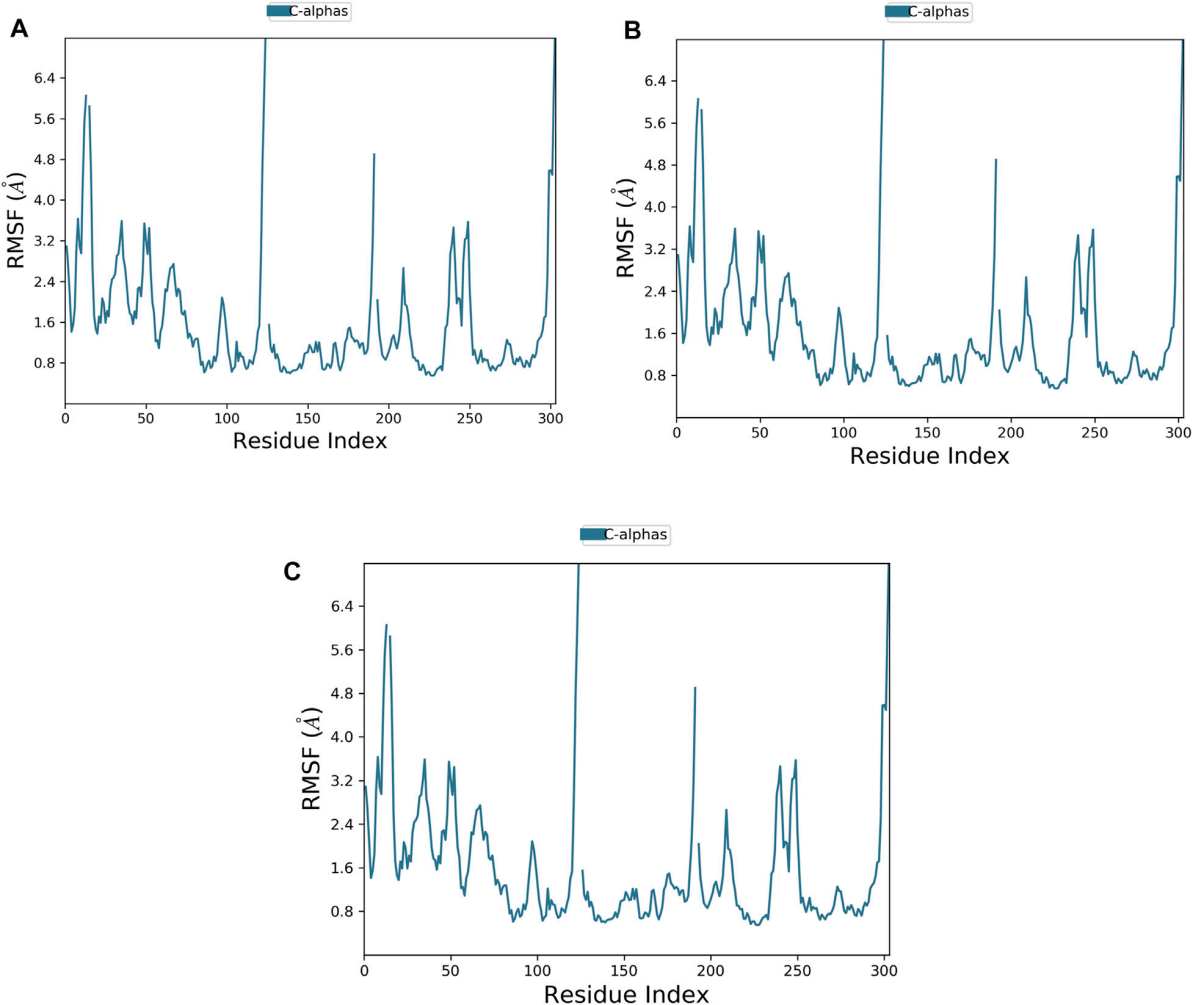
Plot of protein RMSF showing the top three ligands and the reference bound to the TrkA allosteric site (PDB ID: 6D20) during 200 ns molecular dynamics simulation using Desmond software. **(A)** Delanzomib. **(B)** Tibalosin. **(C)** Reference.

The flexibility of the TrkA protein and the movement of its residues were assessed by monitoring the RMSF value of the Cα atoms. A lower RMSF value indicates less flexibility and greater stability (Alzain, 2022). As shown in Figure 4, the protein exhibited similar RMSF patterns with both compounds and the reference, with an average RMSF value of 1.59 Å. The low average RMSF value, combined with the previously discussed average RMSD values, confirms the stability of the studied complexes.

**FIGURE 5:**
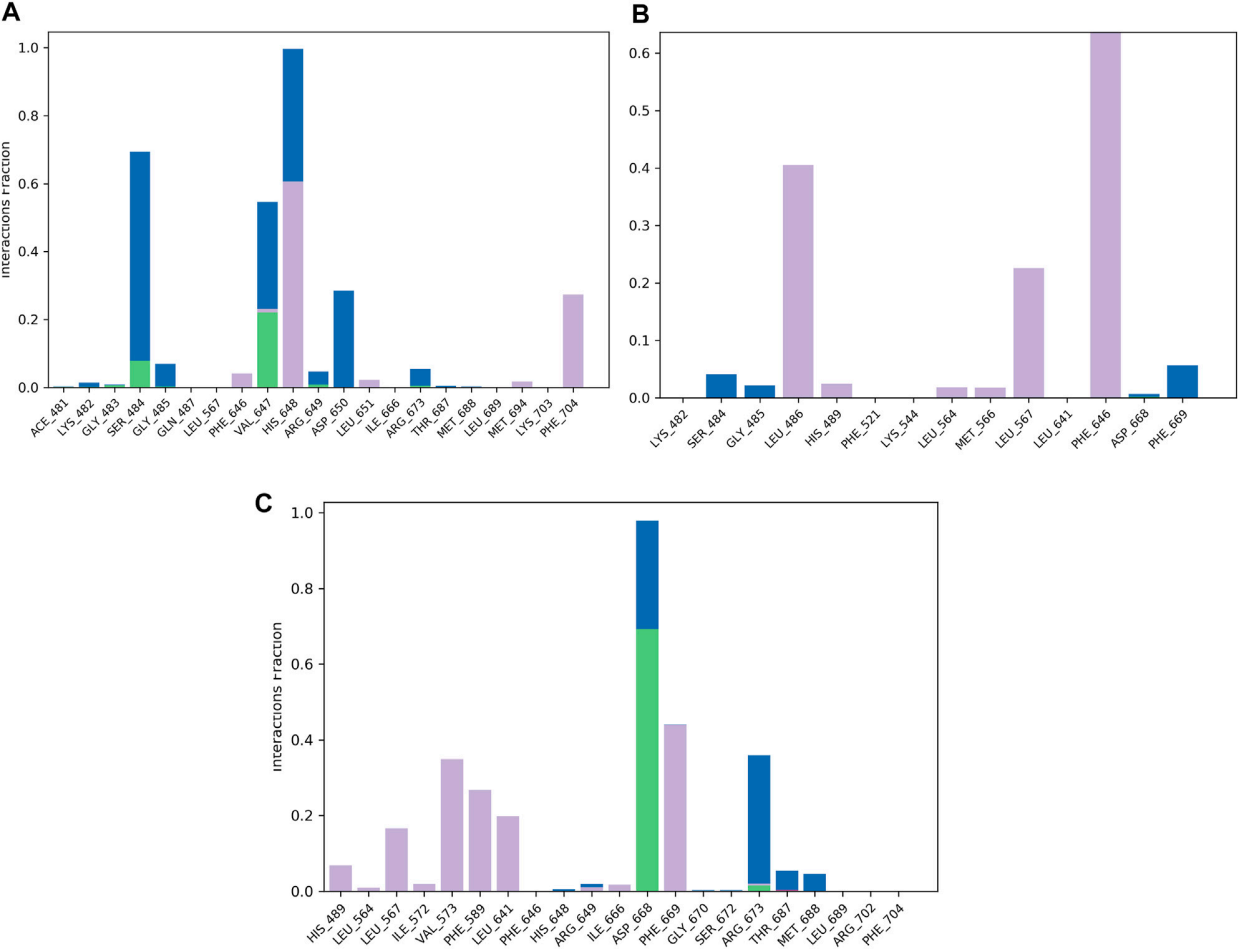
Protein–ligand contact histogram of the top three compounds and the reference complexed with the TrkA allosteric site (PDB ID: 6D20) during 200 ns molecular dynamics simulation using Desmond software. **(A)** Delanzomib. **(B)** Tibalosin. **(C)** Reference.

The protein–ligand contact histogram (Figure 5) provided information about the binding and non-binding interactions between the protein and the two compounds, as well as the reference, during the simulation. The delanzomib–TrkA complex formed contacts with SER484 (H-bond 10% and water bridges 50%), VAL647 (H-bond 25%, hydrophobic 3%, and water bridges 22%), HIS648 (hydrophobic 60% and water bridges 40%), ASP650 (water bridges 25%), and PHE704 (hydrophobic 30%).

The tibalosin–TrkA complex interacted with LEU486 (hydrophobic 40%), LEU567 (hydrophobic 20%), PHE646 (hydrophobic 70%), and PHE669 (water bridges 5%). Considering the significant role of H-bonds in the binding of an inhibitor to a kinase (Wu et al., 2021), these results suggest that delanzomib exhibits higher inhibitory activity than tibalosin. This conclusion is supported by the protein–ligand contact histogram, which shows that delanzomib forms H-bonds with two residues, whereas tibalosin does not form any H-bonds.

The reference–TrkA complex had contacts with LEU567 (hydrophobic 15%), VAL573 (hydrophobic 35%), PHE589 (hydrophobic 30%), LEU641 (hydrophobic 20%), ASP668 (H-bond 70% and water bridge 30%), PHE669 (hydrophobic 45%), and ARG673 (H-bond 2%, hydrophobic 3%, and water bridges 37%).

In conclusion, based on the MD results, delanzomib and tibalosin were identified as type III inhibitors, as they exhibited similar effects on the protein compared to the reference molecule (a potent, selective, and allosteric type III TrkA binder named molecule 23) in terms of RMSD and RMSF plots (Bagal et al., 2019).

## 4 Conclusion

TrkA, the most prevalent isoform associated with a wide range of human malignancies, is a crucial target for cancer therapy. This study aimed to identify potential allosteric TrkA inhibitors for the treatment of cancer. To achieve this objective, multiple computational approaches were employed to screen a library of 9,923 approved drugs from the ChEMBL database, assessing their repurposing potential as allosteric inhibitors against the TrkA protein. Initially, the library was docked into the allosteric site of TrkA using HTVS and XP modes. This screening process yielded 49 compounds with favorable docking scores, which were further evaluated through MM-GBSA calculations to determine their free-binding energies. Among the 49 compounds, nine exhibited MM-GBSA dG bind energies below −50 and were selected for detailed analysis in this study. The interaction patterns of the top two drugs, delanzomib and tibalosin, were examined. These compounds displayed several common interactions with previously identified TrkA allosteric inhibitors, and notably, delanzomib has been reported to possess antimutagenic and anti-cancer effects. Subsequently, delanzomib and tibalosin underwent MD simulations, demonstrating good stability at the protein’s allosteric site. Based on these findings, delanzomib and tibalosin are considered promising hits against TrkA. Further experimental investigations are warranted to validate their potential as inhibitors of this protein, holding significant prospects for future cancer therapies.

## Data Availability

The original contributions presented in the study are included in the article/Supplementary Material; further inquiries can be directed to the corresponding author.

## Abbreviations

TrkA, tropomyosin-receptor kinase A; TrkB, tropomyosin-receptor kinase B; TrkC, tropomyosin-receptor kinase C; NGF, nerve growth factor; Trks, tropomyosin-receptor kinases; RTKs, receptor tyrosine kinases; BDNF, brain-derived neurotrophic factor; NT4/5, neurotrophin-4/5; NT3, neurotrophin-3; ATP, adenosine triphosphate; JM, juxtamembrane; DFG, ASP–PHE–GLY; FFF, PHE–PHE–PHE; CNS, central nervous system; PDB, Protein Data Bank; OPLS, optimized potentials for liquid simulations; FDA, Food and Drug Administration; HTVS, high-throughput virtual screening; SP, standard precision; XP, extra-precision; MM-GBSA, molecular mechanics-generalized born surface area; MD, molecular dynamics; TIP3P, transferable interaction potential; NVT, isothermal–isochoric; NPT, isothermal–isobaric; RMSD, root mean square deviation; RMSF, root mean square fluctuation.
